# Synthesis, Anti-Inflammatory Activity, and In Silico Study of Novel Diclofenac and Isatin Conjugates

**DOI:** 10.1155/2018/9139786

**Published:** 2018-06-12

**Authors:** Musab Mohamed Ibrahim, Tilal Elsaman, Mosab Yahya Al-Nour

**Affiliations:** Department of Pharmaceutical Chemistry, Faculty of Pharmacy, Omdurman Islamic University, Omdurman, Sudan

## Abstract

The design, synthesis, and development of novel non-steroidal anti-inflammatory drugs (NSAIDs) with better activity and lower side effects are respectable area of research. Novel Diclofenac Schiff's bases (**M1, M2, M4, M7, **and** M8**) were designed and synthesized, and their respective chemical structures were deduced using various spectral tools (IR, ^1^H NMR, ^13^C NMR, and MS). The compounds were synthesized via Schiff's condensation reaction and their anti-inflammatory activity was investigated applying the Carrageenan-induced paw edema model against Diclofenac as positive control. Percentage inhibition of edema indicated that all compounds were exhibiting a comparable anti-inflammatory activity as Diclofenac. Moreover, the anti-inflammatory activity was supported via virtual screening using molecular docking study. Interestingly compound** M2** showed the highest in vivo activity (61.32% inhibition) when compared to standard Diclofenac (51.36% inhibition) as well as the best binding energy score (-10.765) and the virtual screening docking score (-12.142).

## 1. Introduction 

Diclofenac is a phenyl acetic acid derivative and belongs to the non-steroidal anti-inflammatory drugs (NSAIDs) family [1].

Despite the huge prescription of NSAIDs, most of them exhibit shared set of adverse effects including gastrointestinal complications [2–6] that are generally attributed to the primary local irritation following the direct contact of carboxylic acid functionality of NSAID with GI mucosal cells and the reduced cytoprotection effect resulting from decreased tissue prostaglandin production [6]; consequently further studies were carried out in order to achieve innovative compounds with lower side effects. In order to attain that goal, many approaches were used including hybridization techniques through linking of new entities or mutual prodrugs [7].

Amide modification of carboxylic acid group of the existing NSAIDs such as Indomethacin, Meclofenamic acid, and Ketoprofen conferred the compounds greater selectivity for COX-2 over the COX-1 enzyme resulting in GI sparing effect [8].

Diclofenac is potent cyclooxygenase enzyme inhibitor with complete absorption and extensive metabolism [9]. Isatin (indoline-2,3-dione) is synthetically versatile substrate serving as raw material for drug synthesis that can be modified into various heterocyclic compounds including Indoles and Quinolones [10]. Comprehensive literature survey revealed that Isatin moiety possesses diverse pharmacological activities including anti-inflammatory activity [9–11]. Further, it has been reported that the substituents at the 2- or 3-position of the indole nucleus are closely related to their anti-inflammatory properties [12–14]. The size of active site in cyclooxygenase enzyme COX-2 is larger than cyclooxygenase enzyme COX-1; hence the extension in ligand size may increase the selectivity toward COX-2 enzyme [15].

In light of the above, regarding the biological importance of Diclofenac and Isatins as well as increasing COX-2 selectivity via extension of ligand size, we aim to design and synthesize novel Diclofenac derivatives with lower side effects, potent activity, and selective cyclooxygenase COX-2 enzyme inhibition. After synthesis, we aimed to evaluate the anti-inflammatory activity via in vivo method and molecular docking study. Many methods are used for the evaluation of anti-inflammatory activity including Carrageenan edema method, which is selected in this essence, as it gives constant results with most of anti-inflammatory drugs besides being rapid and simple in vivo method [16].

Molecular docking is valuable tool for studying the binding affinities and ligand-target interaction [17]; hence we used them for the study and evaluation of anti-inflammatory activity.

## 2. Experimental

### 2.1. Chemistry

The 1H Isatin and 5-bromo Isatin were purchased from Sigma-Aldrich, USA, while Diclofenac was obtained as gift sample from Unamid pharmaceuticals, Sudan.

All solvents and reagents are of analytical grade and used without further purification. Precoated silica gel TLC plates were used to monitor reaction progress; UV lamp (254 nm) was used to visualize the spots. The purity of synthesized compounds was checked by TLC using ethyl acetate and n-hexane (1:1) as a mobile phase. For each compound, a single spot was detected in the TLC plate reflecting the purity of the corresponding compound. Melting points were determined on Electrothermal Karl kolb (scientific technical supplier, Germany) and were uncorrected.

Infrared (IR) spectra were recorded as KBr disk using SHIMADZU FT–IRapparatus, Japan. The data is given in *ύ* (cm-^1^). NMR spectra were determined in acetone and recorded on Bruker Avance 111 NMR Spectrophotometer operating at 400 MHz for ^1^H and at 100 MHz for ^13^C NMR at the research center, College of Pharmacy, Ain Shams University, Egypt. The chemical shifts are expressed as *δ* values (ppm) relative to TMS as internal standard. Signals are indicated by the following abbreviations: s = singlet, bs = broad singlet, d = doublet, dd = doublet of doublet, t = triplet, q = quartet, and m = multiplet. The J constant was given in Hz.

Mass spectra were taken on Single ISQLT Quadrapole–MS spectrometer at the research center, College of Pharmacy, Azhar University, Egypt. Mass spectral data were given as m/z (intensity %).

#### 2.1.1. Procedure for the Synthesis of Intermediate** II**

**Scheme 1:**
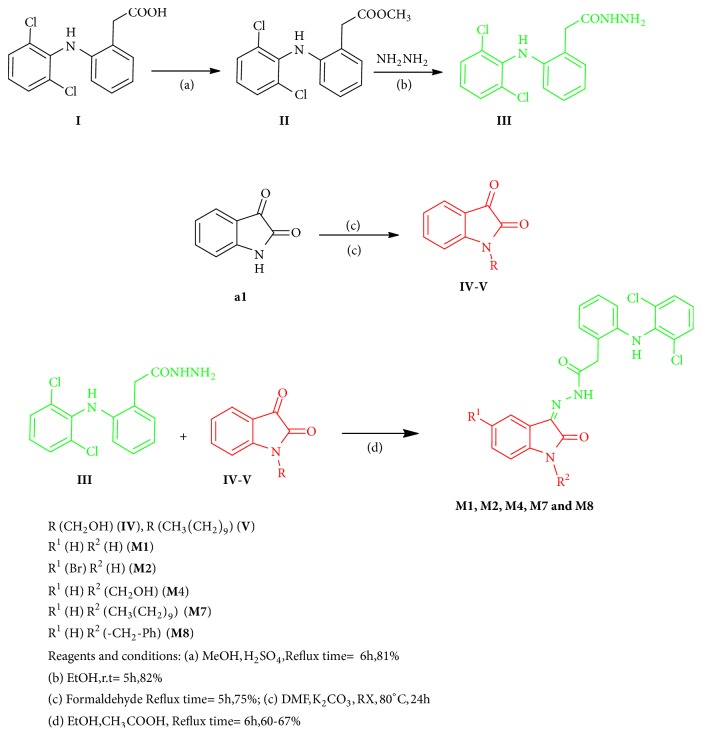
Synthetic route to target compounds.

The esterification reaction was carried out using Fischer esterification procedure (Scheme 1) in acidic media, where 7 grams of Diclofenac acid** I** was weighed and placed in 50 ml round bottom flask and dissolved in 40 ml methanol and few drops of concentrated sulfuric acid were added. The reaction mixture was refluxed for 6 hours and allowed to cool to room temperature. The reaction was monitored by TLC using ethyl acetate and n-hexane (1:1) as mobile phase and the product was obtained by addition of 3 ml of sodium carbonate solution. The product was purified by recrystallization from methanol.

The core reaction is achieved by refluxing equimolar quantities of Diclofenac hydrazide** III** with Isatin nucleus** a1** for 6 hours in Schiff's condensation reaction carried out in the presence of catalytic amount of glacial acetic acid. Preparation of Diclofenac hydrazide** III** involved synthesis of Diclofenac ester** II** from Diclofenac acid** I** via Fischer esterification reaction. The ester** II** is then reacted with hydrazine hydrate to afford the hydrazide** III**. Modification to Isatin nucleus** a1** is carried out either by hydroxy methylation reaction with 37% formalin and refluxing for 5 hours to give intermediate** IV** or through N-Alkylation to afford intermediate** V**.

#### 2.1.2. Procedure for the Synthesis of Intermediate** III**

Five grams of Diclofenac ester (**II**) was placed in 50 ml round bottom flask and dissolved in 20 ml absolute ethanol, and 30 ml of hydrazine hydrate 80% was added (Scheme 1).

The reaction mixture was refluxed for 5 hours. After cooling, 20 ml of distilled water was added; a white precipitate was formed, filtered, dried, and recrystallized from ethanol.

#### 2.1.3. Procedure for the Synthesis of Intermediate** IV-V**

The synthesis of both intermediates is based on N-modification of Isatin** a1** through either hydroxy methylation reaction (**IV**) or N-Alkylation reaction (**V**). For intermediate** IV**, 0.5 grams of appropriate Isatin was initially weighed and refluxed with 20 ml of formalin (37 % v/v) for 5 hours. After evaporation of solvent, the resultant solid was purified by recrystallization from ethanol.

For intermediate** V**, a weight equivalent to 0.03 moles of appropriate Isatin was properly placed in 50 ml round bottom flask containing 20 ml of dimethyl formamide (DMF). An amount equal to 1.5 moles of potassium carbonate was added. This mixture was stirred for 30 minutes; after the specified time, a volume equivalent to 1.5 moles of an alkyl halide was added and the reaction mixture was stirred at 80°C for 24 hours. The reaction was monitored for completion by TLC and final compounds were precipitated by addition of 20 ml of distilled water and then purified by recrystallization from ethanol.

#### 2.1.4. Procedure for the Synthesis of Target Compound** M1**,** M2**,** M4**,** M7**, and** M8**

Equimolar quantities (0.03 moles) of appropriate Isatin** a1** and Diclofenac hydrazide** III** were dissolved in 20 ml of ethanol and refluxed for 6 hours in the presence of 5 drops of glacial acetic acid. Final compounds were obtained and recrystallized from suitable solvent.

The result of yield percent, physiochemical properties, and spectroscopic data are presented in the Results section.

### 2.2. Biological Investigation

The synthesized Diclofenac Schiff's bases were investigated for anti-inflammatory activity via Carrageenan-induced paw edema model. These compounds were tested in a single dose molecularly equivalent to 20mg/Kg of Diclofenac acid and compared to Diclofenac as standard [18, 19].

#### 2.2.1. Experimental Animals

Wistar albino rats of either sex weighing 90-120 grams were obtained from the animal house of Khartoum University, Khartoum, Sudan. All the animals were housed under standard environmental conditions of temperature (24±2°C) and relative humidity of 30-70 %. All the animals were fasted overnight prior to the experiment with free access to water. All the experimental procedures and protocols used were reviewed and approved by the Institutional Animal Ethical Committee (IAEC) of Faculty of Pharmacy, Omdurman Islamic University, constituted in accordance with the guidelines of the Committee for the Purpose of Control and Supervision of Experiment on Animals (CPCSEA).

#### 2.2.2. Chemicals

Carrageenan, carboxymethyl cellulose (CMC), and all the chemicals were of analytical grade.

#### 2.2.3. Protocol

This investigation was performed following procedure of Winter et al. [20]. Animals were divided into 7 groups (n=5). A freshly prepared suspension of Carrageenan (1.0% w/v, 0.1 ml) was injected in the planter region of right hind paw of each rat. Group I served as negative control and only received CMC as vehicle. Group II received Diclofenac 20 mg/kg body weight per oral suspended in 0.5 % CMC. Groups III-VII were treated with the tested compounds, where the dose was molecularly equivalent to Diclofenac and administered orally suspended in 0.5 % of CMC.

One hour after administration of tested compounds, each rat received a subplanter injection of 0.1 ml of a freshly prepared Carrageenan solution (1.0% w/v) in its right hind paw.

The swelling volume of the paw was measured before (time 0) and at 1, 2, 3, and 4 hours after the carrageen injection.

The measurements of the hind paw volumes were given in millimeters (mm) using vernier caliper and the percentage decrease in the paw volume (% inhibition of edema) was calculated from the normal paw volume using the following equation:

(1) $$\begin{array}{c} \%\;\;Inhibition=\frac{Vc-Vt}{Vc}\times 100 \end{array}$$

where Vt is the mean increase in paw volume in rats treated with test compounds and Vc is the mean increase in paw volume in control group of rats [18].

#### 2.2.4. Statistical Analysis

Data was expressed as % inhibition ± S.E.M. and analyzed by one-way ANOVA to determine the significance of the difference between the control group and rats treated with the test compounds. The difference in results was considered significant when P < 0.05. All statistical calculations were carried out using Graph Pad® Prism 5.0 (USA) statistical software [20].

#### 2.2.5. Activity and Lipophilicity

In an attempt to deduce whether lipophilicity affected observed activity of tested compounds, the predicted LogP values of compounds were related to percentage inhibition of oedema as expression of activity. Despite variation in ClogP values, there is no clear relationship between lipophilicity and observed activity as shown in Figure 5.

### 2.3. Computational Analysis

The chemical structure of compounds was drawn via Marven Sketch software version 18.5 [21]. The 3D structure was generated in mol2 with Open Babel software [22], minimized, and optimized with Cresset Flare software [23]. The 3D structure of cyclooxygenase enzyme COX-2 was downloaded from Protein Data Bank [24]. Two structures in good resolution were selected including human COX-2 (PDBID 5IKQ) and* Mus musculus* COX-2 (PDBID 1PXX). For the docking process, the targets were prepared and minimized via Cresset Flare software [23], the grid box was defined according to the clustered ligand of downloaded COX-2 enzymes, and the docking calculations were carried out in Cresset Flare software [23] in normal mode and default settings. Besides the synthesized compounds, Diclofenac, Arachidonic acid, and Rofecoxib were used as control in molecular docking. In order to visualize the 2D and 3D interactions, pose view [25] and Cresset Flare software [23] were used, respectively. The obtained results are shown in Table 2 and Figures 6 and 7.

## 3. Results 

### 3.1. Chemistry

#### 3.1.1. Spectral Data of Intermediates

*Methyl-2-(2-(2,6-dichlorophenylamino)phenyl)acetate** (II)**. *White, odorless powder; Yield%: 82; m.p: 97-99°C [6];** IR **ύ** cm**^−1^** (KBr)**: 3350 sharp and medium (-NH stretch); 3080 (ArH stretches); 2948 (Aliphatic –CH stretch); 1942 (Aromatic overtone); 1735 sharp & strong (C=O stretch); 1587, 1577, 1448(Aromatic C=C stretches); 1292(C-O stretch). ^1^***H NMR-DMSO-d***_6_: *δ* ppm 3.63(s, 3H, -O-C***H***_3_); 3.76 (s, 2H, Ar-C***H***_2_); 6.23(d, 1H, Ar*- ****H***, J=8 Hz); 6.82(td, 1H, Ar-***H***, J=8 Hz, J=1.5 Hz); 7.04(td, 1H, Ar-***H***, J=8 Hz, 1.5 Hz); 7.08(s, 1H, Ar-N***H***-Ar); 7.18(td, 1H, Ar*- ****H***, J=8, 1.5 Hz); 7.20(d, 1H, Ar-***H***, J=8 Hz); 7.53(d, 2H, Ar-***H***, J=8 Hz). ^13^***C NMR-DMSO-d***_6_: *δ* ppm 36.95 (Ar-***C***H_2_); 51.96 (-O-***C***H_3_); 115.66, 120.55, 123, 126.02, 127.77, 129.20, 130.77, 131.01, 137.02, 142.83 (Ar-***C***); 171.9 (***C***=O).** EI-MS, Rel. Int** (%): 310 ([**M**^+^] (100%); 278(15%); 250(16%); 242(11%); 217(13%); 214(60%); 154(30%); 136(23%); 91(12%); 69(17%); 55(20%); 43(19%); 41(15%).

*2-(2-(2,6-Dichlorophenylamino)phenyl)acetohydrazide** (III)**. *Whitish yellow, odorless crystals; Yield%: 81; m.p: 159 - 161°C [27];** IR **ύ** cm**^−1^** (KBr)**: 3325 sharp and strong (-NH stretches); 3022(ArH stretches); 2923 (Aliphatic –CH stretch); 1947(Aromatic overtone); 1637 sharp & strong (C=O stretch); 1598, 1581, 1506(Aromatic C=C stretches).   ^1^***H NMR-DMSO-d***_6_: *δ* ppm 3.50 (s, 2H, Ar-C***H***_2_); 4.32(d, 2H, N-N***H***_2_, J=5 Hz ); 6.29(d, 1H, Ar-***H***, J=10 Hz); 6.84(t, 1H, Ar-***H***, J=5.5 Hz); 7.02(t, 1H, Ar-***H***, J=10 Hz, J=5 Hz); 7.14(t, 2H, Ar-***H***, J=10 Hz, J=5 Hz); 7.51(d,2H, Ar-***H***, J=10 Hz); 8.56(s, 1H, CON***H***); 9.49 (s, 1H, Ar-N***H***-Ar). ^13^***C NMR-DMSO-d***_6_: *δ* ppm 37.68 (Ar-***C***H_2_); 116.06, 120.74, 124.96, 125.37, 127.27, 129.23, 130.35, 137.19, 142.96 (Ar-***C***); 170.85 (***C***=O).** EI-MS, Rel. Int (%):** 310 ([**M+**] (30%); 307(15%); 278(45%); 214(25%); 154(100%); 136(68%); 107(20%); 89(25%); 77(20%); 55(18%); 43(14%); 41(14%).

*1-(Hydroxymethyl) indoline-2,3-dione** (IV)**. *Pale orange, odorless powder; Yield%: 75; m.p: 230-233°C;** IR **ύ** cm**^−1^** (KBr)**: 3446 sharp and medium (-OH stretch); 2983(ArH stretches); 2920(Aliphatic –CH stretches); 1733 sharp & strong(C=O stretches); 1610(Aromatic C=C stretches); 1095(C-O stretch). ^1^**H NMR-Acetone**: *δ* ppm 5.19(s, 2H, N-C***H***_2_); 7.07(td, 1H, Ar-***H***, J=8 Hz, 0.5 Hz); 7.08(d, 1H, Ar-***H***, J=8 Hz); 7.45(dd, 1H, Ar-***H***, J=8 Hz, 0.5 Hz); 7.58(td, 1H, Ar-***H***, J=8 Hz, 1.2 Hz). ^13^**C NMR-Acetone**: *δ* ppm 72.4 (N-***C***H_2_); 111.64, 117.6, 123.6, 124.3, 138.2, 150.7 (Ar-***C***); 157.53(N-***C***=O); 183.6 (***C***=O).** EI-MS, Rel. Int (%):** 177 ([**M+**] (30%); 130(12%); 128(12%);105(14%); 104(25%); 136 (68%); 103(18%); 84(14%); 76(12%); 76(30%); 70(12%); 6 (18%); 65(24%); 64 (14%); 59(12%); 58(52%); 56(24%); 55(48%); 53(30%); 49(30%); 44(72%); 43 (78%); 42(100%).

*1-Decylindoline-2,3-dione** (V)**. *Reddish, odorless powder; Yield%: 68; m.p: 73-75°C;** IR **ύ** cm**^−1^** (KBr)**: 3095(ArH stretches); 2918, 2848(Aliphatic –CH stretches); 1957 (Aromatic overtone); 1737 sharp & strong (C=O stretches); 1608, 1465(Aromatic C=C stretches). ^1^**H NMR-Acetone**: *δ* ppm 0.73(t, 3H, Aliphatic-C***H***_3_, J=8 Hz); 1.13-1.24(m, 12H, Aliphatic-C***H***_2_); 1.56-1.59(m, 2H, Aliphatic-C***H***_2_); 3.62(t, 2H, N-C***H***_2_, J=8 Hz); 7-7.06(m, 2H, Ar-***H***); 7.43(dd, 1H, Ar-***H***, J=7.4 Hz, J=0.68 Hz); 7.55 (td, 1H, Ar-***H***, J=7.8 Hz, J= 1.16 Hz). ^13^**C NMR-Acetone**: *δ* ppm 13.45 (Aliphatic CH3); 22.40, 26.60, 27.06, 31.69, 39.71 (Aliphatic CH2); 61.01(N-CH2-CH2); 72.50(N-***C***H_2_); 110.57, 117.72, 123.16, 124.36, 138.21, 151.34 (Ar-***C***); 157.96 (NH_2_-***C***=O); 183.73(***C***=O).** EI-MS, Rel. Int (%)**: 287 ([**M+**] (2%); 242(12%); 236(10%); 214(20%); 146(12%); 132(12%);130(10%); 117(12%); 111(15%); 109(18%); 98(25%); 97(38%); 95 (30%); 84(38%); 83(55%); 81(42%);73(25%); 71(64%); 69(88%); 67(40%); 60 (18%); 57(100%); 55(92%); 54(15%); 45(22%).

#### 3.1.2. Spectral Data of the Targeted Compounds

*2-(2-(2,6-Dichlorophenylamino)phenyl)-N′-(2-oxoindolin-3-ylidene)acetohydrazide** M1**. *Pale yellow, odorless powder; Yield%: 62; m.p**:** above 240°C;** IR **ύ** cm**^−1^** (KBr):** 3272(NH stretches); 3087, 3072(ArH stretches); 2881, 3823(Aliphatic –CH stretch); 1720 sharp & strong (Ar-NH-C=O stretch); 1666 sharp and strong (-NHN-C=O stretch); 1606(C=N stretch); 1504, 1456 (Aromatic C=C stretches).** EI-MS, Rel. Int (%)**: 439 [**M**^+^] (6%); 307(30%); 289(15%); 154(100%); 136(92%); 107(20%); 89(23%); 77(20%).

*N′-(5-Bromo-2-oxoindolin-3-ylidene)-2-(2-(2,6-dichlorophenylamino)phenyl) acetohydrazide** M2**. *Pale orange, odorless powder; Yield%: 66; m.p: above 240°C;** IR **ύ** cm**^−1^** (KBr)**: 3363(NH stretches); 3143, 3107(ArH stretches); 2846, 2792(Aliphatic –CH stretch); 1915(Aromatic overtone); 1733 sharp & strong (Ar-NH-C=O stretch); 1685 sharp and strong (-NHN-C=O stretch); 1606(C=N stretch); 1494, 1467, 1446 (Aromatic C=C stretches). ^1^**H NMR-Acetone**: *δ* ppm 3.53(s, 2H, Ar-C***H***_2_); 6.42(d, 1H, Ar-***H***, J=7.4 Hz); 6.92(s, 1H, Ar-***H***); 7.18(d, 1H, Ar-***H***, J=7.4 Hz); 7.46-7.48 (m, 6H, Ar-***H***); 8.03(s, 1H, Ar-***H***).** EI-MS, Rel. Int (%)**: 518 ([**M**^+^] (22%); 516(12%); 281(20%); 280(34%); 279(78%); 276(100%); 244(20%); 242(70%); 241(42%); 239(38%); 215(15%); 214(44%); 181 (20%); 179(28%); 151(22%); 132(12%); 88(12%); 77(12%).

*2-(2-(2,6-Dichlorophenylamino)phenyl)-N′-(1-(hydroxymethyl)-2-oxoindolin-3-ylidene)acetohydrazide** M4**. *Pale red, odorless powder; Yield%: 64; m.p: above 240°C;** IR **ύ** cm**^−1^** (KBr)**: 3267 Broad (OH stretch); 3033(ArH stretches); 2972(Aliphatic CH_2_ stretch); 1911(Aromatic overtone); 1787 sharp & strong (Ar-NH-C=O stretch); 1622 sharp and strong (-NHN-C=O stretch); 1612(C=N stretch); 1583, 1504, 1452 (Aromatic C=C stretch). ^1^**H NMR-Acetone**: *δ* ppm 3.62(s, 2H, Ar-C***H***_2_-); 4.14(s, 2H, N-C***H***_2_-OH); 6.45(d, 1H, Ar-***H***, J=8 Hz); 6.87-6.88(m, 1H, Ar-***H***); 7.11-7.13(m, 4H, Ar-***H***); 7.30(m, 2H, Ar-***H***); 7.43-7.46(m, 3H, Ar-***H).***

^13^ **C** **NMR-Acetone:** *δ* ppm 61.04(Ar***C***H_2_-C=O); 72.4(N-***C***H_2_-OH); 116.4, 117, 121.22, 121.29, 123.61, 123.78, 124.39, 124.60, 125.39, 127.34, 127.48, 129.02, 129.05, 129.51, 129.56, 129.61(Ar-***C***); 158(-***C***=O-NH); 170.44 (***C***=O).

**EI-MS, Rel. Int (%)**: 469 ([**M**^+^] (2%); 410(22%); 408(70%); 406(100%); 392 (18%); 381(20%); 380(50%); 378(75%); 366(20%); 364(30%); 363(15%); 344 (20%); 343(20%); 333(50%); 291(32%); 289(50%); 256(38%); 254(28%); 217 (25%); 216(28%); 213(90%); 117(10%); 107(55%); 106(20%); 95(12%); 83 (12%); 71(15%).

*N′-(1-Decyl-2-oxoindolin-3-ylidene)-2-(2-(2,6-dichlorophenylamino)phenyl)acetohydrazide** M7**. *Pale yellow, odorless powder; Yield%: 67; m.p: above 240°C;** IR **ύ** cm**^−1^** (KBr)**: 3288(NH stretches); 3066(Ar-H stretches); 1722 sharp & strong (Ar-NH-C=O stretch); 1664 sharp and strong (-NHN-C=O stretch); 1610(C=N stretch); 1510, 1458(Aromatic C=C stretches). ^1^**H NMR-Acetone**: *δ* ppm 0.71(t, 3H, Aliphatic-C***H***_3_, J=7 Hz); 1.21-1.58(m, 4H, Aliphatic-C***H***_2_); 2.81(s, 1H, Ar-N***H***-Ar); 3.47(s, 2H, Ar-C***H***_2_); 3.68(t, 2H, N-C***H***_2_, J=7 Hz); 4.236(s, 1H, R-C=O-N***H***); 6.33(d, 1H, Ar-***H***, J=8 Hz); 6.77(t, 1H, Ar-***H***, J=7.3 Hz); 6.99-7.03(m, 4H, Ar-***H***); 7.26-7.33(m, 4H, Ar-***H***); 7.64(d, 1H, Ar-***H***, J=7.3 Hz).

^13^ **C** **NMR-Acetone:** *δ* ppm 13.48(Aliphatic***C***H_3_); 29.01(Aliphatic***C***H_2_); 29.8(Aliphatic***C***H_2_); 39.39(N-CH_2_-***C***H_2_); 62(C=O-***C***H_2_); 72.51(N-***C***H_2_); 117.07, 119.71, 120.66, 121.47, 123.07, 124.89, 127.66, 128.98, 129.05, 129.85, 129.91, 131.38, 131.54, 134.18, 137.69(Ar-***C***); 160.99(NH-***C***=O);173.98(N-***C***=O).

**EI-MS, Rel. Int (%)**: 579 ([**M**^+^] (1%); 302(40%); 301(100%); 285(40%); 278(30%); 277(18%); 225(15%); 224(62%); 214(20%); 130(15%); 128(18%); 105(15%); 103 (48%); 102(25%); 89(10%); 77(25%); 75(50%); 74(20%); 69(18%); 57(14%).

*N′-(1-Benzyl-2-oxoindolin-3-ylidene)-2-(2-(2,6-dichlorophenylamino)phenyl)acetohydrazide** M8**. *Pale yellow, odorless powder; Yield%: 67; m.p: above 240°C;** IR **ύ** cm**^−1^** (KBr)**: 3288(NH stretches); 3199 (Ar-H stretches); 2966, 2920(Aliphatic CH_2_ stretches); 1726 sharp & strong (Ar-NH-C=O stretch); 1664 sharp and strong (-NHN-C=O stretch); 1606(C=N stretch); 1508, 1460(Aromatic C=C stretches). ^1^**H NMR-Acetone**: *δ* ppm 3.60(s, 2H, Ar-C***H***_2_-C=O); 3.98(s, 2H, Ar-C***H***_2_-N); 6.3-7.35(m, 11H, Ar-***H***); 7.53(dd, 2H, Ar-***H***, J=8 Hz, J=4 Hz); 8.20(s, 2H, Ar-***H***).

### 3.2. Biological Activity

**Figure 1:**
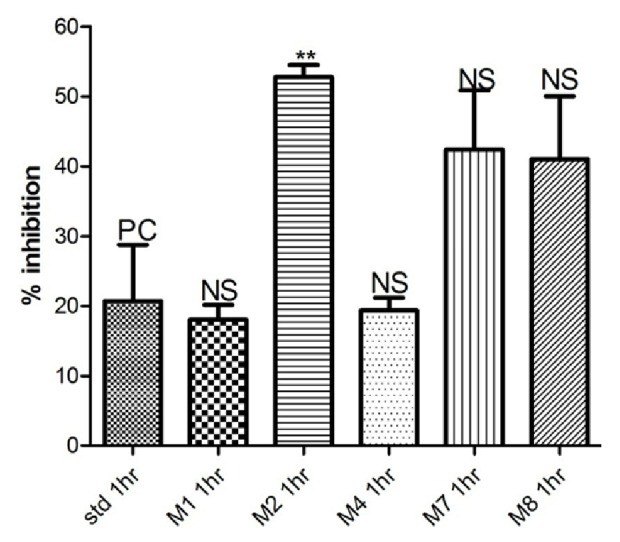
Anti-inflammatory activity in the first hour.

**Figure 2:**
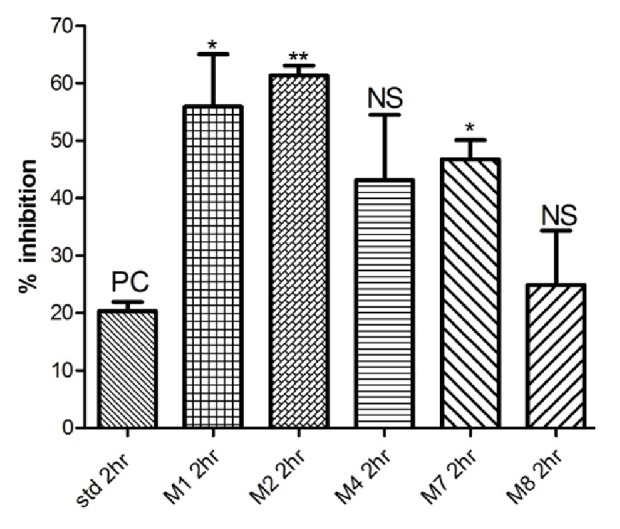
Anti-inflammatory activity in the second hour.

**Figure 3:**
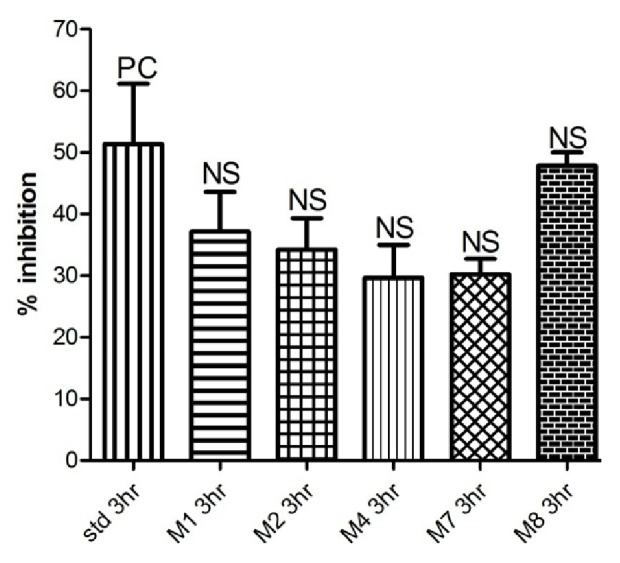
Anti-inflammatory activity in the third hour.

**Figure 4:**
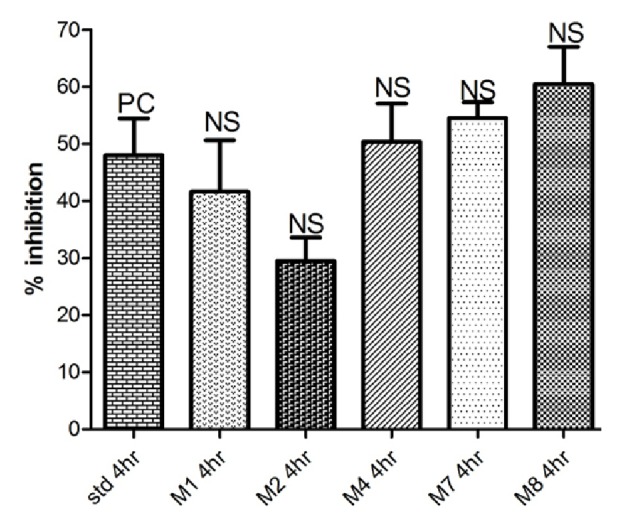
Anti-inflammatory activity in the fourth hour.

**Figure 5:**
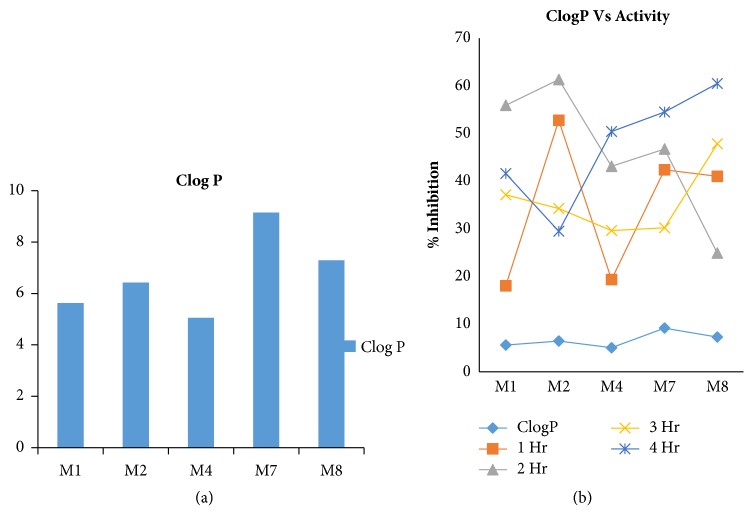
Lipophilicity and activity. (a) ClogP values of the synthesized compounds; (b) % inhibition and lipophilicity.

See Table 1 and Figures 1–5.

### 3.3. Molecular Docking Study

**Figure 6:**
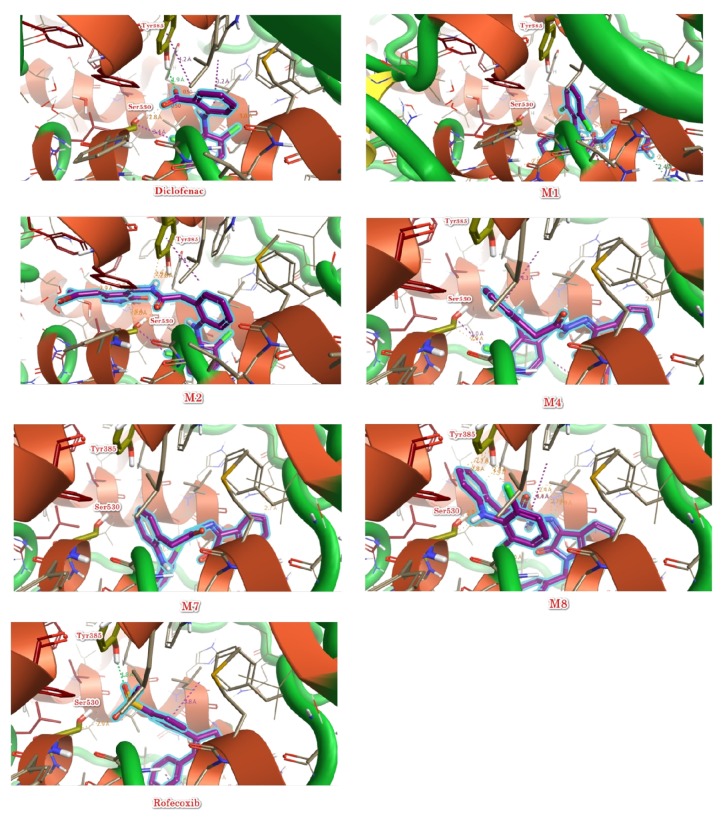
3D binding modes and interactions of the synthesized compounds with COX-2 enzyme. The compounds are illustrated in violet colour. Diclofenac and Rofecoxib are used as control compounds.

**Figure 7:**
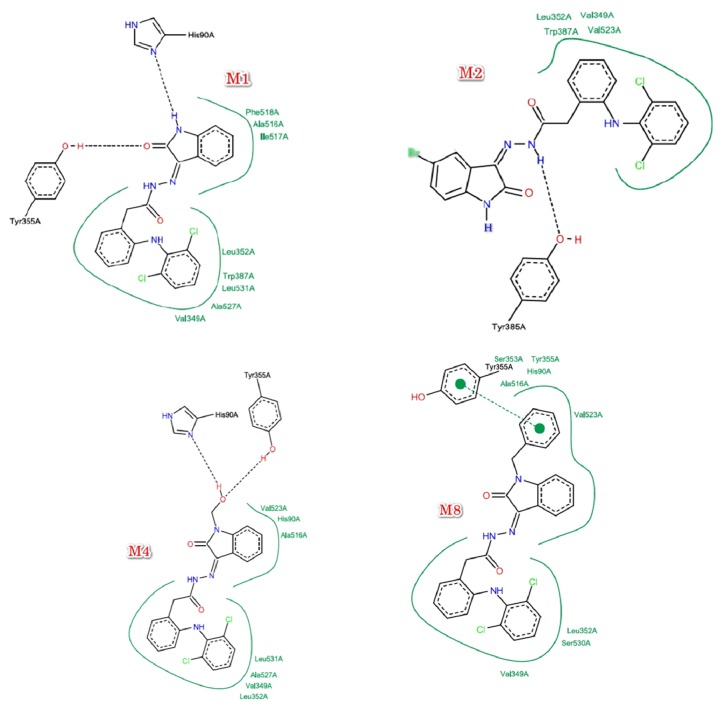
2D interactions between the synthesized compounds and COX-2 enzyme.

See Table 2 and Figures 6 and 7.

## 4. Discussion 

**Figure 8:**
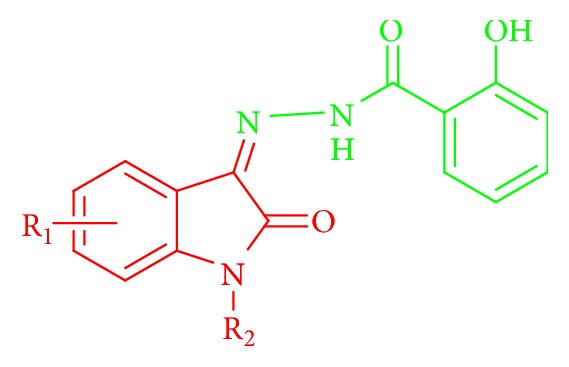
Promising anti-inflammatory compounds were synthesized by condensation of salicylic acid carbohydrazide with substituted Isatin.

The development of new anti-inflammatory drugs via hybridization techniques through linking of new entities or mutual prodrugs has been used extensively in literature to obtain new compounds with better actions [7]. It is reported that a promising anti-inflammatory compounds were synthesized by condensation of salicylic acid carbohydrazide with substituted Isatin (Figure 8) [28]. In continuation of our efforts to develop safe and effective anti-inflammatory compounds [29, 30], we designed and synthesized new Diclofenac-Isatin hybrids and screened them for in vivo anti-inflammatory activity with molecular docking studies.

Spectral characterization tools (IR, NMR, and MS) were applied to confirm the proposed structures of synthetic compounds, alongside physical data such as TLC analysis and melting point. The IR spectra of Diclofenac Schiff's bases were clearly differentiated from their respective intermediates, evidenced by absence of ketone stretching (1730 cm^−1^) and appearance of a band in the range of 1585-1620 cm^−1^ which denotes stretching of the newly formed imine functional group (C=N). Furthermore, the spectra bear two major sharp and strong absorption bands between 1720-1780 cm^−1^ and 1660-1690 cm^−1^ characterizing stretches of two carbonyl functional groups (C=O lactam) and (C=O hydrazide), respectively. For ^1^H NMR, the most observable trend is related to aromatic protons that were always equal to the sum of aromatic protons of target compounds. The compounds also shared a common absorption signal around 3.55 ppm, characterizing the Diclofenac aliphatic methylene group (Ar-C***H***_2_-). The ^13^C NMR spectra of** M4** and** M7** are generally featured by increase in aromatic carbons compared to corresponding starting materials indicating the success of the reaction. Similarly, the spectra showed a signal around 72 ppm recognizing the (N-***C***H_2_-) moiety and two carbonyl absorptions at around 160 ppm and 170 ppm relating to the cyclic carbonyl group (N-***C***=O) and the acyclic carbonyl group (Ar-CH_2_-***C***=O), respectively. Although the molecular ion peak [**M**^+^] varied in intensity, it was evidenced for all analyzed Schiff's bases. A special peak at* m/z *= 215 seemed to form a common fragment among the compounds.

The Carrageenan-induced paw edema is a useful in vivo model to evaluate the contribution of mediators involved in vascular changes associated with acute inflammatory response following induction of inflammation using inflammatory inducing agent [27]. From Carrageenan-induced paw edema model data, we found that the synthesized compounds,** M1** and** M2**, illustrated a higher activity than** M4**,** M7**, and** M8**.

Among the synthesized compounds,** M2 **showed the best and fastest activity (61.32% inhibition) (Table 1 and Figures 1–4).

In Carrageenan model, the development of edema in the rat hind paw following the injection of Carrageenan is a biphasic response, including the initial phase of edema (0-1 hour) that is not inhibited by NSAIDs, as well as the second phase of swelling (1-6 hours) that involve production of prostaglandins from the inducible cyclooxygenase (COX-2) enzyme [27]. Within the synthesized compounds,** M1** and** M2** onset of action seems to be earlier than other synthesized compounds that start at the first hour and maximize in the second hour, indicating that** M1** and** M2 **act on the late responses to inflammation. The pattern of activity of compound** M4** is rather different from other candidates; the compound showed activity (43.13% and 50.39%) at two different time intervals (second and fourth hours, respectively). In contrast, in** M7** and** M8**, the maximum inhibition (54.52% and 60.48%, respectively, that was almost similar to activity of** M1** and** M2**) was achieved at the fourth hour, suggesting that they have a delayed action. This delay in onset of action might be explained by the high lipophilicity that leads to distribution out of blood, or as they are prodrugs that metabolized via N-Dealkylation into the active forms (**M1). **

**Figure 9:**
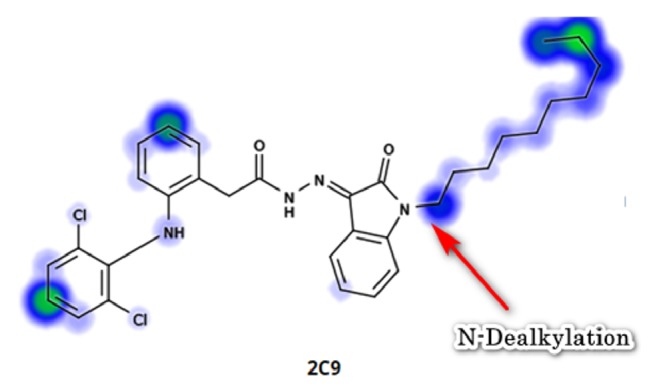
**M7** metabolism prediction.

In order to validate this hypothesis, we used XenoSite server [31] to predict the metabolism of compounds. The result showed that the compounds are susceptible to N-Dealkylation via Cytochrome 2C9 enzyme (Figure 9).

The pharmacological activity of NSAIDs is linked to the suppression of prostaglandin biosynthesis from arachidonic acid through inhibiting the enzyme cyclooxygenases [4]. In order to support the results of in vivo study, molecular docking study was executed. According to the correct energy ranking (Rank score), correct rank ordering (VS-score), and binding energy predictions (Dg-score) [26], the compounds** M2 **and** M4** were the best and this finding is in consistence with the in vivo results.

Moreover, all of the synthesized compounds having a binding energy score (Dg-score) are better than the control compounds (Diclofenac, Arachidonic acid, and Rofecoxib) (Table 2). Regarding** M2**, it showed the best binding energy score (Dg-score) and the virtual screening docking score, but due to the 2 violations of Lipinski rule-of-five (Rof5) it showed a decreased Rank score. On the contrary,** M4** has no violations in Lipinski rule-of-five (Rof5); consequently it showed the highest Rank score (Table 2).

The most important amino acids in active site of cyclooxygenase (COX) enzymes are Tyrosine (Tyr385) that is responsible for the activity via hydrogen abstraction and radical formation as well as Serine (Ser530); hence formation of hydrogen bond between NSAIDs and Tyr385 will inhibit the activity of cyclooxygenase (COX) enzymes and the formation of hydrogen bond with Ser530 will lead to irreversible inhibition [15]. According to 3D interaction results,** M2 **forms hydrogen bonds with Tyr385 and Ser530. Additionally, the binding conformation of** M2 **is identical to Diclofenac, meaning that** M2 **will be promising cyclooxygenase (COX) enzyme inhibitor with irreversible action; consequently** M2 **is proven to be the best of synthesized compounds (Figure 6).

**Figure 10:**
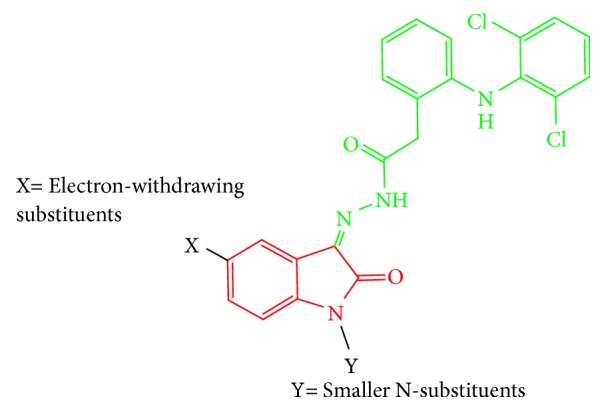
The proposed SAR of tested compounds.

At the level of the Structure Activity Relationships (SAR), we noticed that the hydrogen bond acceptors are essential for binding with the oxygen atom of hydroxyl group in amino acid Tyr385 (the essential amino acid for the activity [15]) and hence are essential for the activity (e.g., in the case of Diclofenac, the hydrogen bond acceptor is hydrogen of carboxylic acid group and in the case of compound** M2**, it is the hydrogen of secondary amino group in amide bond). Moreover, we noticed that the aromatic system in Diclofenac is important for the hydrophobic interaction with the hydrophobic amino acids in the active site; hence it is important for stabilization of binding conformation. Furthermore, the neutral amide bond between Diclofenac and Isatin moiety masked the acidic functional group of Diclofenac that serve in reduction of gastrointestinal side effects. In addition, electron withdrawing groups at para position of phenyl ring in Isatin moiety (as in** M2**) prevent the interaction of Isatin's phenyl group (as in** M1**) that alter the optimum binding conformation leading to reduction in activity** (M2 **is better than** M1) **(Figure 7). On the other side, activity showed by** M4**,** M7**, and** M8 **is less than** M1** and** M2**. This difference in activity would be influenced by the type of substituent in N-position of Isatin nucleus, pointing that the smaller the substituent in N-position, the higher the resultant activity, whereas bulky N-substituents such as benzyl and decyl groups cause steric clashes that decrease the effective binding to receptors, hence resulting in lower activity. The general SAR of tested compounds is shown in Figure 10.

## 5. Conclusion

Five Diclofenac Schiff's bases (**M1, M2, M4, M7, **and** M8**) were designed and their chemical structures were spectrally confirmed. The compounds were evaluated in vivo and in silico for their possible anti-inflammatory activity. The 5-bromo substituted compound (**M2**) showed the maximum in vivo activity and scored the best binding energy and virtual screening docking scores, arriving at the fact that it could be a good candidate for anti-inflammatory drug of higher activity, COX-2 enzyme selectivity, and lower gastrointestinal side effects. It is suggested that compounds** M7** and** M8 **are prodrugs evidenced by their delayed onset of action and their susceptibility to N-Dealkylation by Cytochrome 2C9 enzyme.

## Figures and Tables

**Table 1:** Anti-inflammatory activity of tested compounds at a dose molecularly equivalent to 20mg/Kg Diclofenac.

**Compound**	**Percentage inhibition at different time intervals (inhibition ± SEM)**			
	**1 hour**	**2 hours**	**3 hours**	**4 hours**
Diclofenac	20.71 ± 8.0	20.34 ± 1.5	51.36 ± 9.7	47.99 ± 6.4
**M1**	18.05 ± 2.1	55.90 ± 9.1 *∗*	37.12 ± 6.4	41.61 ± 9.0
**M2**	52.75 ± 1.7 *∗∗*	61.32 ± 1.7 *∗∗*	34.23 ± 5.0	29.47 ± 4.1
**M4**	19.35 ± 1.8	43.13 ± 11.3	29.63 ± 5.3	50.39 ± 6.6
**M7**	42.40 ± 8.4	46.76 ± 3.3 *∗*	30.23 ± 2.4	54.52 ± 2.8
**M8**	41.03 ± 8.9	24.91 ± 9.4	47.83 ± 2.1	60.48 ± 6.5

*Data analyzed by one way ANOVA followed by Dunnett's test (n=5). ∗P<0.05 and ∗∗P<0.01.*

**Table 2:** The correct energy ranking (Rank score), virtual screening docking score or correct rank ordering (VS-score), and binding energy predictions (Dg-score) [26] of the docked compounds with COX-2 enzyme 3D structures (5IKQ and 1PXX) as well as Lipinski rule-of-five violations (Rof5).

**Compound**	**5IKQ**				**1PXX**		
	**Rank score**	**VS-score**	**dG**	**Rof5**	**Rank score**	**VS-score**	**dG**
M1	-5.875	-10.339	-8.092	0	-6.889	-11.055	-10.140
M2	-5.305	-12.142	-10.765	2	-6.455	-12.855	-10.112
M4	-7.170	-11.710	-8.732	0	-6.828	-11.347	-9.974
M7	-4.755	-11.041	-8.241	2	-6.740	-12.504	-12.690
M8	0.277	-7.115	-4.666	2	-6.015	-12.767	-10.715
Diclofenac	-9.319	-9.885	-9.549	0	-9.069	-9.962	-9.403
Arachidonic Acid	-8.581	-10.508	-9.063	1	-8.476	-9.545	-8.913
Rofecoxib	-8.237	-10.097	-8.241	0	-8.061	-9.708	-8.538

## Data Availability

The data used to support the findings of this study are available from the corresponding author upon request.
